# Comparison of interscalene block, general anesthesia, and intravenous analgesia for out-patient shoulder reduction

**DOI:** 10.1007/s00540-019-02624-6

**Published:** 2019-03-12

**Authors:** Janett Kreutziger, Desmond Hirschi, Sven Fischer, Richard F. Herzog, Stefan Zbinden, Philipp Honigmann

**Affiliations:** 1 Department of Anesthesiology and Critical Care Medicine, Medical University of Innsbruck, Anichstr. 35, 6020 Innsbruck, Austria; 2 Private Practice, Worb, Switzerland; 3 Department of Anesthesiology and Intensive Care Medicine, Muri Regional Hospital, Muri, Switzerland; 4 Department of Orthopedics and Trauma Surgery, Lucerne Cantonal Hospital, Wolhusen, Switzerland; 5 Department of Anesthesiology and Intensive Care Medicine, Lucerne Cantonal Hospital, Wolhusen, Switzerland; 6 Department of Orthopedic Surgery, Cantonal Hospital of Basel-Land, Liestal, Switzerland

**Keywords:** Shoulder dislocation, Interscalene block, General anesthesia, Patient satisfaction

## Abstract

**Purpose:**

Shoulder dislocation is often associated with intense pain, and requires urgent pain therapy and reduction. Interscalene block, general anesthesia, or intravenous analgesia alone are applied procedures that facilitate shoulder reduction by the surgeon and ease patients’ pain. This study was conducted to compare procedure times, patient satisfaction, side-effects, and clinical outcome of these clinical procedures.

**Methods:**

Retrospective chart analysis was performed for all patients treated at the Emergency Department of a primary care hospital. In addition, standardized telephone interviews were conducted. Subjective clinical outcome and patient satisfaction (SF-36, Quick-DASH, ZUF-8) were measured with the standardized questionnaires.

**Results:**

The shortest overall procedure time [67.5 min (48.8–93.5 min), *P* = 0.003] was found in patients with interscalene block. The advantage of general anesthesia was the shortest anesthesia induction time [10 min (7.8–10 min), *P* < 0.0001]; reduction time [6 min (4.3–6 min), *P* = 0.039]; and time to discharge [90 min (67.5–123.8 min), *P* = 0.0001] were significantly prolonged in comparison to interscalene block [5 min (1–5 min) and 45 min (2–67.5 min)]. The longest reduction time [11 min (10–13.5 min), *P* = 0.0008] was seen in patients in the intravenous analgesia group. Overall, patient satisfaction was greater in patients with regional as compared to general anesthesia [measured by ZUF-8: 12 (9–15) vs. 17 (12–24), *P* = 0.03]. Subjective clinical outcome (SF-36, DASH) was comparable among the three groups. There was one immediately identified esophageal intubation in the general anesthesia group.

**Conclusions:**

Out-patient shoulder reduction can be accomplished no matter whether general anesthesia, regional anesthesia, or intravenous analgesia alone was administered. Clinical outcome as measured by SF-36 and DASH was comparable among the three groups, but the shortest overall procedure time and greater patient satisfaction were found in patients with interscalene block.

## Introduction

Shoulder dislocation is often associated with intense pain and requires urgent and efficient analgesic therapy. General or regional anesthesia (interscalene block), or intravenous analgesia alone are alternate, applied procedures that allow the surgeon to reduce the shoulder dislocation easily and free of or with little pain to the patient [1–5]. To date, comparative data for these three methods are scarce. Therefore, this study aimed to compare these methods regarding overall procedure time, patient satisfaction (measured with a questionnaire: German-language version of the Client Satisfaction Questionnaire CSQ-8 [6, 7], and pain rated on the numerical rating scale, NRS [8]) and clinical outcome as measured with two standardized questionnaires: short SF-36 (German-language version) [9] and Quick-DASH (disability arm–shoulder–hand) [10]. Both evaluate the subjective allocation of health status regarding the shoulder and the extent of limitation of everyday life. In addition, any side-effects should be recorded.

The null hypothesis was that there is no difference in outcome parameters. Alternative hypothesis was that overall procedure time is shorter and overall patient satisfaction is greater with interscalene block, whereby clinical outcome is comparable among the three groups.

Primary outcome was overall procedure time. Secondary outcome parameters were patient satisfaction (measured with ZUF-8), course of pain, number of side-effects or any adverse event, and subjective clinical outcome as measured with SF-36 and Quick-DASH.

## Materials and methods

Following approval by the Ethics Committee of Canton Lucerne, Switzerland, a retrospective data analysis was performed for all adult patients (≥ 18 years) admitted to the Emergency Department of a primary care hospital in Switzerland for shoulder dislocation. The staff consisted of four anesthesiologists and four specialists in orthopedics and traumatology, and one last-year trainee in each specialization. The study took place from January 1, 2004 to August 30, 2010. Inclusion criterion was a radiologically confirmed diagnosis of shoulder dislocation following trauma. Exclusion criteria were incomplete data records or refusal to participate in the study.

Beside demographic and morphometric data, also reported were profession, anamnesis for earlier dislocations and pre-existing diseases which were charted. In addition, side and type of dislocation (anterior, posterior), accompanying injuries (Bankart and Hill-Sachs lesions, fractures, neurological, and other), type of surgical procedure (Davos, modified Hippocrates, and Kocher method) [11], co-administration of analgesic drugs, type of anesthesia, type and dose of drugs, several procedure times, time of immobilization, and inability to work, all complications possibly related to anesthesia or surgical procedures. During the study period, there were no guidelines specifying the type of anesthesia/analgesia or the drugs and their dosages. Both anesthetic techniques have been routinely used and mastered by all the members of the anesthetic team for years. However, an anamnestic high-grade chronic obstructive pulmonary disease would have been a relative contraindication for an interscalene block.

### Anesthesia/analgesia

Normally, all patients received 1000 mg of acetaminophen and intravenous morphine, as required, soon after arrival at the hospital. All patients undergoing general or regional anesthesia were continuously monitored by ECG, pulse oximetry, and oscillatory blood pressure measurement. Intravenous access was obligatory. Interscalene block was either ultrasound guided (out-of-plane technique) or performed with a combination of ultrasound and nerve stimulation under sterile conditions. Normally, 10–20 ml prilocaine 1% or 10 ml of prilocaine 1% and ropivacaine 1% each were administered. Maximum volume did not exceed 20 ml. Routinely, no additional sedative drugs were administered during regional anesthesia.

General anesthesia was initiated by intravenous administration of short-acting narcotics and analgesic drugs, mask ventilation, and orotracheal intubation, if necessary. In patients who received intravenous analgesia alone, no anesthetist was involved in patient management.

Patients were discharged to home when awake, fully oriented, circulatory-stable, post-interventional peripheral oxygen saturation was similar to pre-interventional without additional oxygen, without newly occurred hoarseness (paresis of phrenic or recurrent nerve); when patients denied nausea or vomiting, also following drinking of water or tea, they had one successful micturition and the post-surgical pain level was acceptable to the patient. Patients had regular out-patient follow-ups with the treating surgeon. If necessary, neurological assessment was performed by a neurologist.

### Procedure times

Procedure times were defined as follows: anesthesia induction time was defined as the time of presence of an anesthetist up to surgical start of shoulder reduction. Reduction time was defined as surgical time from start-up to successful completion of shoulder reduction. Time from completion of the shoulder reduction to discharge included anesthetic weaning and observation of vital parameters up to initial values, administration of brace, radiological check-up, and so on. Overall procedure time was the sum of all three times.

### Patient satisfaction

All patients were systematically interviewed by two trained persons (authors JK, DH) with the standardized ZUF-8 questionnaire (German-language version of the Client Satisfaction Questionnaire CSQ-8). The questionnaire consists of eight questions (items); each provides a Likert-type scale with four response levels numbered 1–4. Agreements have low level numbers (1, 2) and disagreements have higher level numbers (3, 4). The overall satisfaction is reflected by the sum of the level numbers; hence, the lower the number, the greater the patient satisfaction [6, 7].

### Pain

Pain scores according to the numeric rating scale (NRS, 0–10) [8] were routinely asked and documented at different time points at the Emergency Department (on admission, following administration of pain drugs, during and following reposition, at discharge). Pre-hospital pain scores were documented in protocols, as well. In addition, patients were also asked about pain intensity during the first evening.

### Safety

Any side-effect or adverse event was charted.

### Clinical outcome

All patients were systematically interviewed by two trained persons (authors JK, DH) using the following standardized questionnaires: short SF-36 (German-language version) [9], Quick-DASH (disability arm-shoulder-hand) [10], which measures subjective conditions regarding the affected shoulder and limitations in everyday life caused by the affected shoulder.

### Statistics

The study population and questionnaires were analyzed by descriptive statistics following the Kolmogorov–Smirnov test for normal distribution. Due to the retrospective design of the study and missing pilot data, no power analysis could be performed. Potential confounding influence or risk factors such as age, gender, pre-existing diseases, accompanying injuries, drug administration, and of type of reduction were evaluated using the Mann–Whitney *U* test, Chi-square test, and Fisher’s exact test, as appropriate. Correlation was done with Pearson’s or Spearman’s correlation test, as appropriate. A significance level of 0.05 was used throughout the study. All results were shown as medians and interquartile range.

## Results

During the study phase, 67 patients with a confirmed diagnosis of shoulder dislocation were admitted to the emergency department. Four patients were excluded: one because he refused to participate in a telephone interview, two were unavailable for months by telephone, and one was homeless and impossible to reach by telephone. Of these 63 patients, 31 received an interscalene block, 18 general anesthesia, and 14 intravenous analgesia alone for shoulder reduction (Table 1). Among the patients who underwent regional or general anesthesia, there was no one with pre-existing recurrent nerve paralysis or severe chronic obstructive pulmonary disease.

**Table 1:** Characteristics of study population *n* = 63

Median (interquartile range) or number (%)	Interscalene block (*n* = 31)	General anesthesia (*n* = 18)	Intravenous analgesia (*n* = 14)	*P* values
Age	57 (52.5–72.5)	57 (43–60)	38.5 (30.8–63.8)	0.49
Gender female/male	10/21 (32.3%/67.3%)	3/15 (16.7%/83.3%)	4 / 8 (33.3%/66.7%)	0.44
Body mass index	23.8 (21–25.4)	25.7 (24.2–27.7)	25.8 (24.5–26.9)	0.94
Patients with habitual dislocations	4 (12.9%)	3 (16.7%)	1 (7.1%)	0.79
Type of reduction Kocher/Davos/Hippocrates/spontaneous	4 (12.9%)/10 (32.3%) 13 (41.9%)/4 (12.9%)	2 (11.1%)/7 (38.9%) 9 (50.0%)/0	0/11 (78.6%) 2 (14.3%)/1 (7.1%)	< 0.0001
Drugs used predominantly				
	100 mg ropivacaine 1% + 100 mg priclocaine 1%	100–200 mg propofol 1% + 1 mg alfentanil (100 mg succinylcholine)	75 mg diclofenac iv + 1000 mg acetaminophen iv + 2–10 mg morphine iv	

Linear results are presented as medians and interquartile range in brackets; binomial results are presented as numbers and percent in brackets

Patients in the general anesthesia group with a fasting time of less than 6 h were orotracheally intubated, which was necessary for four (22.2%) patients; they received succinylcholine as the sole muscle relaxant. Although, in many cases, the fasting time was not documented, there was no case of aspiration in any group.

Except for the particular reduction technique, patient characteristics were comparable among the three groups. There was a trend to younger patients in the group receiving intravenous analgesia alone, but high variance within groups prevented this from reaching statistical significance (Table 1).

### Procedure time

Anesthesia induction time was the shortest in the general anesthesia group [10 min (7.8–10 min), *P* < 0.0001], but reduction time [6 min (4.3–6 min), *P* = 0.039] and time from completion of reduction to discharge [90 min (67.5–123.8 min), *P* = 0.0001] were prolonged in comparison to interscalene block [23 min (15–27.5 min), 5 min (1–5 min), 45 min (26–67.5 min)]. The longest reduction time [11 min (10–13.5), *P* = 0.0008] was seen in patients in the group receiving intravenous analgesia alone. The shortest overall procedure time from induction of anesthesia/analgesia to discharge was seen in patients with interscalene block [67.5 min (48.8–93.5 min), *P* = 0.003] (Table 2).

**Table 2:** Overall procedure time including time of anesthesia induction, reposition, and to discharge of all patients, *n* = 63

Times (*t*) in min as median (interquartile range)	Interscalene block (*n* = 31)	General anesthesia (*n* = 18)	Intravenous analgesia (*n* = 14)	*P* values ISB vs. GA	*P* values ISB vs. IVA	*P* values GA vs IVA
*t* Anesthesia induction to start of reduction	23 (15–27.5)	10 (7.8–10)	25 (18.8–30)	< 0.0001	0.5	0.0005
*t* Reduction	5 (1–5)	6 (4.3–6)	11 (10–13.5)	0.039	0.0008	0.075
*t* Completion of reduction to discharge	45 (25–67.5)	90 (67.5–123.8)	70 (56.3–112.5)	0.0001	0.049	0.44
Overall procedure time	67.5 (48.8–93.5)	102 (100–110)	125 (102.5–147.5)	0.003	0.0007	0.25

Results are presented as medians and the interquartile range in brackets

*ISB* interscalene block, *IVA* intravenous analgesia, *GA* general anesthesia

### Patient satisfaction

Overall, patient satisfaction as measured with the ZUF-8 was greater in patients with regional as compared to general anesthesia [median sum: 12 (9–15) vs. 17 (12–24), *P* = 0.03]. There was no significant difference in overall patient satisfaction between the interscalene block group and the intravenous analgesia group [median sum: 12 (9–15) vs. 11 (8–13), *P* = 0.22] despite higher pain levels in the latter. Patients who had previously undergone anesthesia stated that they would prefer regional instead of general anesthesia (*P* = 0.047) or intravenous analgesia alone (*P* = 0.039). General anesthesia was much preferred in comparison to intravenous analgesia alone (*P* = 0.01). The patient’s experience with regard to shoulder reduction was more favorable for interscalene block than for general anesthesia (*P* = 0.033) or intravenous analgesia (*P* = 0.036). Nevertheless, for shoulder reduction, most patients (52/63; 83%) stated that they would choose the same anesthetic procedure they had previously undergone for any surgery, if ever (no group differences).

### Pain

The initial pain scores (pre-hospital, at admission, following administration of pain drugs) were comparable among the three groups (Table 3). In 12 (19%) of 63 patients, shoulder reduction was attempted pre-hospital, but was not successful. Instead, patients suffered severest pain. During in-hospital reduction, patients with intravenous analgesia alone [NRS 3.5 (0–4)] experienced more intense pain than did patients with interscalene block [0 (0–0), *P* = 0.02] or general anesthesia [NRS 0 (0–0), *P* = 0.002]. Administered morphine equivalents during procedure [35 (5–50), *P* < 0.0001] and overall [40 (16–53.5), *P* = 0.003] were highest in patients with general anesthesia. The lowest overall amount of morphine equivalents was achieved with interscalene block [6 (3.5–8.5), *P* ≤ 0.003] (Tables 3, 4).

**Table 3:** Pain intensity at different time points as measured by numeric rating scale

Median (interquartile range)	Pre-hospital	On admission	After pain drugs	During reduction	On discharge	First evening
Interscalene block	9 (6–9)	9 (2–9)	8 (0–8)	0 (0–0)	0 (0–0)	0 (0–0)
General anesthesia	10 (0–10)	6.6 (3–7)	6 (3–6.5)	0 (0–0)	2 (0–2.5)	2.5 (0–3)
Intravenous analgesia	10 (5–10)	8 (4–9)	5 (3–7)	3.5 (0–4)	2 (0–2)	2 (0–2)
*P* values (ISB vs. GA)	0.53	0.063	0.50	0.89	0.04	0.006
*P* values (ISB vs. IVA)	0.52	0.45	0.11	0.02	0.002	0.008
*P* values (GA vs. IVA)	0.94	0.40	0.17	0.002	0.67	0.83

Results of pain intensity evaluation according to the NRS (numeric rating scale). Results are presented as medians and the interquartile range in brackets

*ISB* interscalene block, *IVA* intravenous analgesia, *GA* general anesthesia

**Table 4:** Analgesic drugs as a function of anesthetic procedure *n* = 63

Median (interquartile range)	Interscalene block (*n* = 31)	General anesthesia (*n* = 18)	Intravenous analgesia (*n* = 14)	*P* values ISB vs. GA	*P* values ISB vs. IVA	*P* values GA vs. IVA
Morphine equivalents pre-procedure	6 (3.5–8.5)	4.2 (2.3–5.8)	6 (4.5–7.5)	0.13	0.37	0.054
Morphine equivalents during procedure	0 (0–0)	35 (5–50)	5 (3.8–6.2)	< 0.0001	< 0.0001	0.12
Morphine equivalents post-procedure	0 (0–0)	0 (0–0)	1.5 (0.8–2.3)	0.65	0.066	0.33
Morphine equivalents overall	6 (3.5–8.5)	40 (16–53.5)	12.5 (9.8–14.3)	0.003	0.001	0.048

Results are presented as medians and the interquartile range in brackets

*ISB* interscalene block, *IVA* intravenous analgesia, *GA* general anesthesia

### Clinical outcome

The majority (39/63; 61.9%) of patients stated that their shoulder function had deteriorated after shoulder dislocation and that they were still suffering from persisting pain in the affected shoulder [general anesthesia 15/18; 83.3%, regional anesthesia 18/31; 58.1%, intravenous analgesia alone 6/14; 42.9%; (*P* = 0.04)]. Accordingly, patients were still experiencing different sensations over the affected shoulder than over the healthy shoulder [general anesthesia 4/18, 22.2%, regional anesthesia 7/31, 22.5%; intravenous analgesia alone 8/14; 57.1%, (*P* = 0.04)]. Nevertheless, limitations when performing several activities (carrying bags, hanging up washing, hammering, cutting foods, etc.) showed no differences among the groups. Time of immobilization was comparable among the groups (around 21 days); the duration of inability to work differed nominally, but did not reach statistical significance [interscalene block: 41.5 days (21.5–98); general anesthesia 60 days (19.2–99.3), and intravenous analgesia alone 42 days (31.5–49)].

In seven (7/63; 11.1%) patients, sensomotor deficits were documented on admission to the emergency department; three patients (3/63; 4.8%) patients were still suffering from persistent sensory deficits of the arm. According to neurological evaluation by a neurological specialist, in all three cases, the neurological deficits were trauma sequelae. Systemic diseases such as diabetes mellitus, alcohol abuse, chemotherapy, etc. were excluded as the cause of these deficits. No association with patient age, gender, body mass index, anamnesis for preceding dislocations, side and kind of dislocation, type of anesthesia, or surgical procedure with the measured clinical outcome (SF 36, Quick-DASH) was found.

### Patient safety

There was one immediately identified esophageal intubation in the general anesthesia group without any sequelae. There was no case of aspiration. With regard to methemoglobinemia, no patient showed a relevant decrease in peripheral oxygen saturation following prilocaine administration (maximum 200 mg). No Horner syndrome was documented following interscalene block and there was no reason for a delayed discharge. There were no signs of a relevant paresis of phrenic or recurrent nerve (hoarseness and measurement of peripheral oxygen saturation). No other side-effect or adverse event was documented in any of the patients. Three (3/63; 4.8%) patients were hospitalized. These included two following general anesthesia (one due to persisting pain and the other for further therapy due to the complexity of the injury). An additional patient was hospitalized for social reasons following regional anesthesia (single, age 75 years, unable to care for herself while immobilized).

## Discussion

In this small, retrospective, single-center study in patients with traumatic shoulder dislocation, regional anesthesia (interscalene block), general anesthesia, and intravenous analgesia alone were feasible and safe procedures for pain therapy and shoulder reduction. However, slight differences were found: a higher postoperative pain level and prolonged time to discharge following general anesthesia and intravenous analgesia alone. The main advantages of a single-shot, ultrasound-guided interscalene block were effective pain relief and faster shoulder reduction as well as shorter time to discharge. Overall patient satisfaction was greater in patients undergoing regional anesthesia, although most patients stated that, for shoulder reduction, they would choose the same anesthetic procedure which they had previously undergone for any surgery, if ever. The type of anesthesia/analgesia did not influence patients’ clinical outcome.

Population characteristics and pre-hospital pain levels were comparable to those of other studies [2, 12–14] and did not differ significantly among the treatment groups except for significantly more reductions according to the Davos method and a trend towards younger patients in the group with intravenous analgesia alone (Table 1). The anesthetic and surgical procedures used during the study period have been described in the literature [1, 4, 5, 12, 14].

### Procedure times

Provision of regional anesthesia to patients for shoulder reduction led to a longer delay until the surgeon could perform shoulder reduction as compared to general anesthesia. The longer duration was due to the time needed from onset to complete muscle relaxation. Therefore, in this situation, the use of prilocaine, an agent with a short onset of action [12], appears preferable. In addition, prilocaine has the pharmacological advantage of having less neurotoxic effects than, e.g., lidocaine [15], which could be an alternative local anesthetic with a short onset of action and which has been used for out-patient shoulder reduction [2, 4, 5, 14]. In contrast, the significantly shortest reduction time was seen in the patients with regional anesthesia; one explanation could be the more profound muscle relaxation in comparison to that in patients with general anesthesia (mostly without muscle relaxant) or intravenous analgesia alone. Furthermore, patients were able to leave the hospital shortly after shoulder reduction without needing time to recover from anesthesia.

The choice of general anesthesia provided the shortest delay until the surgeon was able to reduce the shoulder dislocation (Table 2). This is not surprising, because administration of short-acting narcotics and opioids with or without muscle relaxants provides good conditions for reducing the shoulder immediately. Reduction times were identical in patients with muscle relaxants and in those without [with: 5 min (4.75–8.75 min), without: 5 min (4.25–5 min), *P* = 0.32], but the number of patients was too small to draw a significant conclusion. Following shoulder reduction, patients remained hospitalized on average for one and a half hours until discharge after recovering from anesthesia.

In contrast, intravenous analgesia alone entailed longer anesthesia induction and reduction times, as well as time to discharge. This study confirmed the results reported by Michael Blaivas and Raeyat Doost [5, 14], who found prolonged hospital stay for shoulder reduction following analgosedation in comparison to interscalene block. The surgeon’s reasoning when deciding to use intravenous analgesia alone instead of calling for an anesthetist may be the lack of delay as compared to waiting for an available anesthetist, maybe, hoping for a short overall procedure time and that the procedure can be performed with fewer drugs. However, the above results confirm the opposite despite the fact that most patients underwent reduction using the Davos method, which is known to be relatively gentle. Furthermore, patients indicated pain and discomfort during and following the reduction.

### Patient satisfaction

Patient satisfaction was quite high in all the groups, but statistically significantly greater satisfaction was seen for regional anesthesia. According to sub-questions on the ZUF-8, this rural population had a preference for regional anesthesia, biasing overall patient satisfaction. However, the currently available literature shows greater patient satisfaction for regional anesthesia than for general anesthesia [16, 17]. This greater patient satisfaction often accompanies lower pain levels, which we also observed in our population, especially post-interventional levels.

For shoulder reduction, the methods of Kocher, modified Hippocrates, and Davos were chosen by the surgeons. For immobilization, most patients received a Don-Joy stabilizer, but there were also several patients with Gilchrist and Orthogilet braces. There was no difference in patient satisfaction in connection with the type of reduction or immobilization.

### Pain

In the group with intravenous analgesia alone, relevant amounts of morphine equivalents were necessary and several patients suffered considerable pain during and after reduction, especially in comparison to patients with regional anesthesia (Tables 3, 4), which is why the authors consider this type of peri-interventional accompanying therapy to be not the best choice. However, despite higher pain levels, patients were quite satisfied with this method and there were no complaints.

### Clinical outcome

Patients were examined pre-interventionally for sensomotor deficits, but, from the point of view of the authors, especially motor function may be deemed to be restricted as it is overlaid by severest pain. During the acute situation in the emergency department, it may be hard to assess whether rapid shoulder reduction would improve sensomotor function, which was true in four (4/7; 57%) out of seven patients, or if the deficit might be permanent. Therefore, the decision as to which kind of anesthesia should be used in a particular patient was not influenced by the presence or absence of sensomotor deficits or dysesthesias. The authors wish to underline the fact that no patient showed an accident mechanism or clinical signs of a suspected plexus lesion, which would be a contraindication for regional anesthesia.

All patients were examined post-interventionally as out-patients and at fixed intervals by a traumatologist. If necessary, a neurologist also saw the patients. All persistent neurological deficits were diagnosed as trauma sequelae. The relatively high percentage of all patients with pain and different sensations over the affected shoulder cannot be explained by any factor analyzed in this trial. Despite these different sensations, patients were not limited to a greater extent than patients without these problems.

### Patient safety

In this small-study population, no relevant complication was detected, and other than one immediately identified esophageal intubation, no other adverse event was observed. No patient showed a relevant decrease in peripheral oxygen saturation following prilocaine administration. Thus, a relevant methemoglobinemia may be excluded as far as possible with the use of maximum 200 mg prilocaine in adult patients with the correct interscalene blockade. Consequently, all three procedures may be considered for shoulder reduction, but ultrasound-guided, single-shot interscalene block appears to have the advantages of being a faster procedure and having greater patient satisfaction.

### Limitations

The limitations of this study are the small patient number, the single-center design, and the retrospective character with some time delays between the event and the patient’s telephone interview. This delay may have colored the patient’s degree of satisfaction. Indeed, most patients were able to remember the events in detail and describe their pain—especially that suffered during pre-hospital reduction maneuvers—in surprising detail. Patients were seen to have a clear bias regarding their satisfaction: they were involved in the decision about the type of anesthesia they preferred for shoulder reduction. As this was true for all patients, their satisfaction with their choice was probably high.

The large percentage of patients with persisting pain and limitations in everyday life should prompt more research for improvements in the interdisciplinary therapy of shoulder dislocations.

## Conclusion

Out-patient shoulder reduction can be accomplished no matter whether general anesthesia, regional anesthesia, or intravenous analgesia alone was administered. Clinical outcome as measured by SF-36 and DASH was comparable among the three groups, but the shortest overall procedure time and greater patient satisfaction were found in patients with interscalene block.
